# From Chemical Burn to Below Knee Amputation: Amputation Secondary to Application of Over-the-Counter Topical Analgesic With Menthol and Methyl Salicylate

**DOI:** 10.7759/cureus.20191

**Published:** 2021-12-06

**Authors:** Rohan Desai, Jesus Gutierrez, Sundar V Cherukuri, Jesus Guzman, Abhizith Deoker

**Affiliations:** 1 Internal Medicine, Texas Tech University Health Sciences Center El Paso, El Paso, USA

**Keywords:** below-the-knee amputation, methyl salicylate, menthol, chemical burn, topical analgesic

## Abstract

Topical analgesics and topical rubefacients are widely used to control acute as well as chronic pain every day. Due to their availability without a prescription, consumers often overlook any potentially harmful effects and consider them largely benign. Here, we present a rare case of chemical burn triggered by a typical over-the-counter (OTC) analgesic containing menthol and methyl salicylate resulting in chemical burn, complicated by necrotizing infection treated by below the knee amputation.

## Introduction

Topical analgesics encompass a wide array of formulations that are used every day for acute and chronic pain management. Osteoarthritis and neuropathic pain management guidelines increasingly recommend their use due to their safety profile and over-the-counter (OTC) availability; however, these agents are not without a folly [1,2]. Food and Drug Administration (FDA) issued a safety announcement to the public about rare cases of serious burns associated with the use of OTC topical pain relievers containing the active ingredients menthol, methyl salicylate, or capsaicin. We present a rare case of such an adverse drug event further complicated by necrotizing soft tissue infection requiring below-the-knee (BKA) amputation.

## Case presentation

A 47-year-old Hispanic male with a past medical history of uncontrolled type 2 diabetes mellitus and diabetic neuropathy was admitted to the hospital for worsening subacute right lower extremity (RLE) pain and erythema with new purulent drainage. The patient first visited the emergency department two weeks prior for RLE pain and erythema without drainage that he had noticed after applying a commonly used OTC topical ointment containing methyl salicylate with menthol. He had started applying the ointment in the morning each day for relief of muscle aches and was leaving it for the entire day. During the initial visit, the patient was afebrile with mild sinus tachycardia at 101 beats per minute (BPM) and blood pressure (BP) of 125/69. On examination, there was circumferential erythema with pitting edema and fluid-filled blisters. Labs during this visit were remarkable for alkaline phosphatase (ALP) of 132 and hemoglobin A1c of 12.5%. RLE x-ray and Doppler ultrasonogram (US) were obtained for further investigation and to rule out deep vein thrombosis, respectively. The imaging studies were remarkable only for soft tissue edema. After several hours of observation, the patient was discharged with triamcinolone for contact dermatitis and outpatient follow-up. However, worsening white cell count and concern for infection led to re-evaluation in the emergency department and subsequent hospitalization. 

**Figure 1:**
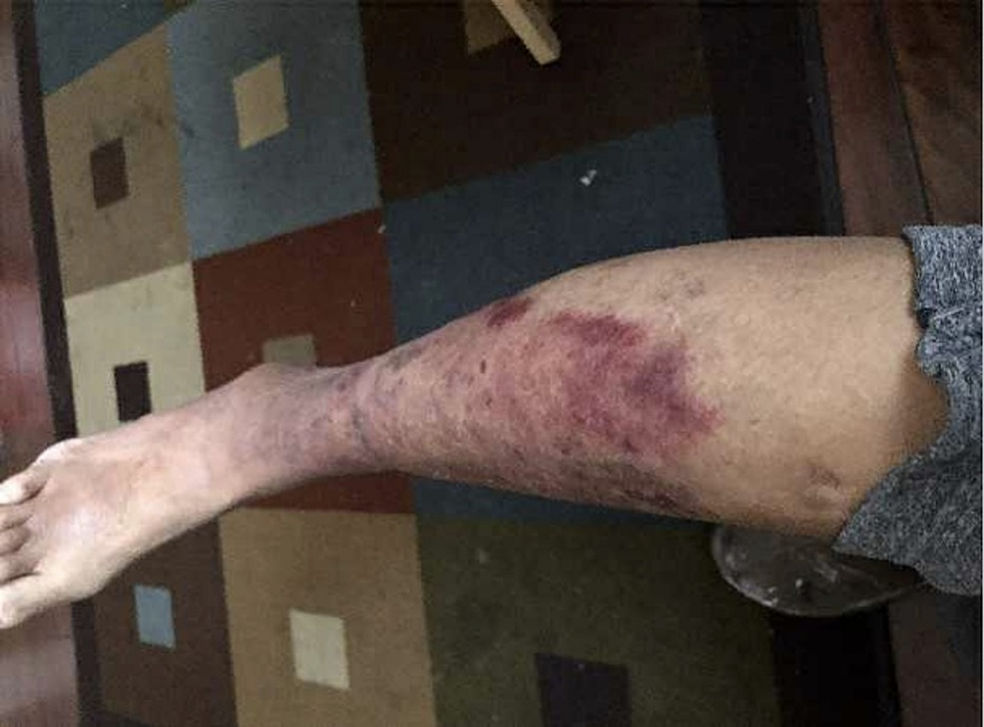
Right lower extremity erythema likely from chemical burn.

**Figure 2:**
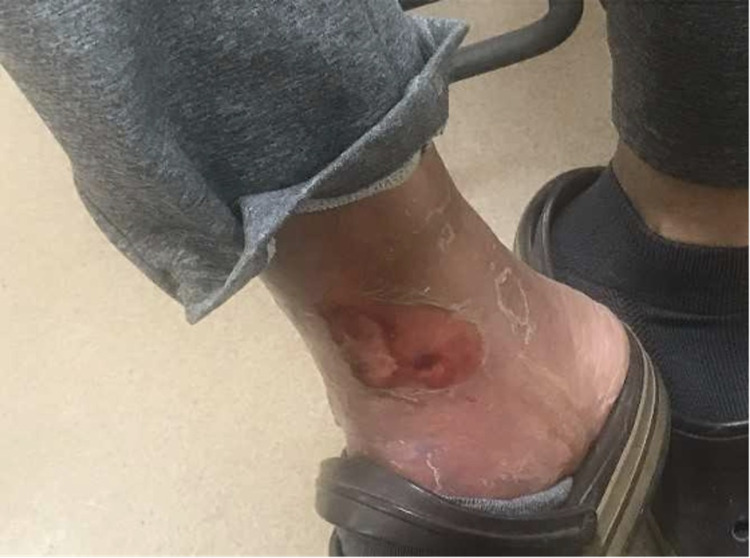
Right lower extremity open wound likely after a blister.

**Figure 3:**
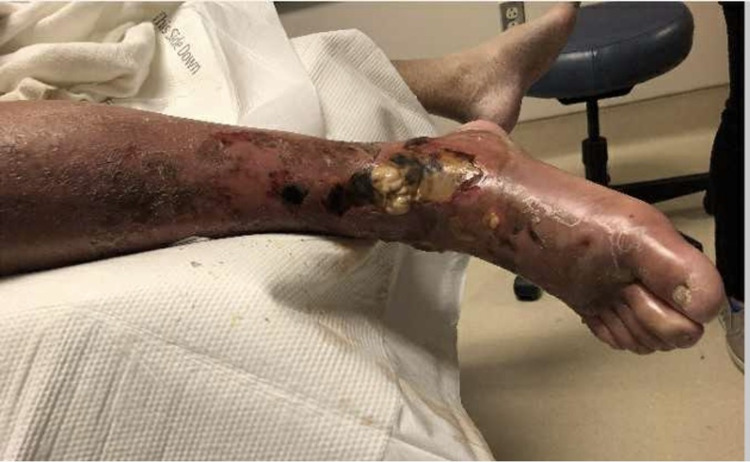
Right lower extremity with purulent drainage on admission.

**Figure 4:**
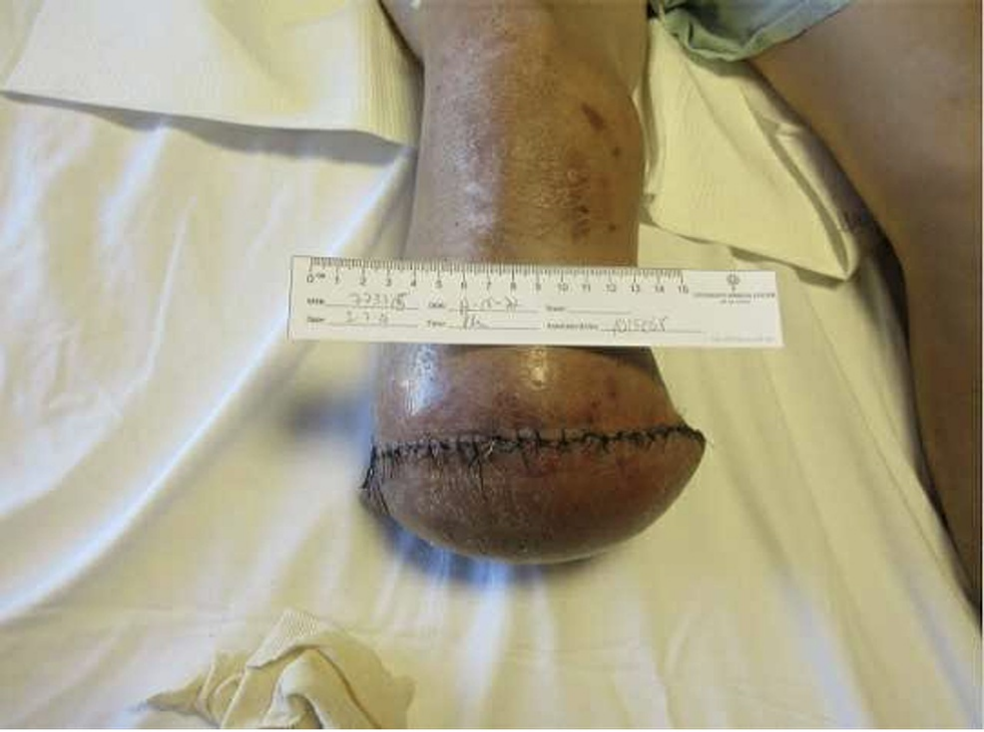
Status post below-the-knee amputation.

On admission, the patient was afebrile, with a heart rate of 93 BPM, and a BP of 138/68. Physical examination was remarkable for necrotic skin changes to the dorsum of the right foot with draining pustules (Figures 1-3). He was admitted to the surgical intensive care unit service with a diagnosis of right lower extremity necrotizing skin and soft tissue infection. Admission labs were remarkable for WBC 8.4, erythrocyte sedimentation rate (ESR) of 120, C-reactive protein (CRP) >18, and serum creatinine of 1. RLE x-ray indicated soft tissue infection with subcutaneous emphysema without osteomyelitis. He underwent RLE below the knee amputation on the day of admission and intra-operative pathology specimen findings were remarkable for skin with ulceration with associated soft tissue abscess and necrosis, chronic osteomyelitis, and extensive deep soft tissue abscess formation (Figure 4). Surprisingly, the patient had never had a diabetic foot ulcer or amputation prior to this hospitalization. 

## Discussion

Topical analgesics are medications applied to the skin or mucous membranes for a variety of acute and chronic painful conditions. Topical analgesics is a broad umbrella term that encompasses a variety of medications including nonsteroidal anti-inflammatory drugs (NSAIDs), rubefacients, capsaicin, and lidocaine. Topical NSAIDs act by locally inhibiting cyclooxygenase enzyme and decreasing the synthesis of prostanoids. As there is limited systemic absorption, the risk of upper gastrointestinal bleeding is low with chronic use of topical NSAIDs [3]. Rubefacients are skin irritants that act by causing dilation of blood vessels resulting in increased blood flow leading to the sensation of warmth and redness of the skin. It acts as a counter-irritant by causing irritation of sensory nerve endings, which alters the pain sensation carried by the same nerves. Salicylates are typically considered rubefacients, not NSAIDs, and were a component of the OTC analgesic used by our patient [4]. Capsaicin acts by blocking nociceptors but is often preceded by a burning sensation. Lidocaine decreases peripheral nociceptor sensitization and central nervous system hyperexcitability by stabilizing the neural membrane [4].

Topical medications are absorbed through the skin and exert their effect locally with minimal systemic uptake as opposed to transdermal medications which act by systemic absorption [5]. Due to the lack of systemic absorption, topical analgesics avoid several systemic side effects and are typically considered safer compared to transdermal or oral formulations. Due to the safety profile and OTC availability, topical analgesics are increasingly recommended in osteoarthritis, especially the knee, and neuropathy pain management guidelines [1,2,6-8].

Despite their safer profile, these agents can still cause significant harm if not used as recommended. In 2012, the FDA issued a safety announcement to the public about rare cases of serious burns ranging from first to third-degree chemical burns associated with OTC products containing menthol, methyl salicylate, and capsaicin. Forty-three such cases were identified and second and third-degree burns were more common in products containing menthol or menthol and methyl salicylate, especially when the concentration of menthol or methyl salicylate was greater than three or ten percent, respectively [9].

A chemical burn can be caused by acids or bases and result in liquefactive or coagulative necrosis, respectively, and is influenced by a variety of factors including pH, concentration, volume, and length of time, the agent is applied [10]. In our patient, the application of OTC topical medication for prolonged duration caused chemical burn and due to the diabetic neuropathy, the patient was unable to appreciate common symptoms such as irritation or burning. Unfortunately, the patient's course was further complicated by the development of necrotizing soft tissue infections (NSTI), characterized by necrotic infection to the skin, subcutaneous tissue, and superficial fascia. NSTI typically develops after skin breakdown serves as an entry point and the lower extremities are the most common site [11]. One of the main risk factors for NSTI is diabetes mellitus and most likely served a crucial role in the development of superimposed infection [11]. NSTI is associated with high morbidity and mortality, often requiring surgical intensive care unit admission. Treatment involves broad-spectrum antibiotics and eventually surgery, such as BKA, for source control. 

Despite the FDA safety warning, the existing tentative final monograph does not require labels warning consumers that the use of the products could result in serious burns [9]. While the patient’s progression from applying OTC analgesics to BKA is extremely rare, this case highlights the importance of medication reconciliation of even the most benign medications. It is imperative that physicians counsel patients on chemical burns as a possible side effect and educate them on the proper method of use.

## Conclusions

Topical menthol and menthol plus methyl salicylate are common components of various over-the-counter muscle and joint pain medications and are often self-prescribed. This case highlights the importance of medication reconciliation in every patient encounter regardless of the setting and the dangers of overlooking commonly prescribed OTC medication interactions.
